# Diaspore Dimorphism, Awn Hygroscopicity and Adaptive Significance in a Winter Annual *Bromus tectorum* (Poaceae)

**DOI:** 10.3390/plants13213093

**Published:** 2024-11-03

**Authors:** Jiayue Yan, Qian Li, Bo Zhang

**Affiliations:** 1 College of Grassland Science, Xinjiang Agricultural University, Urümqi 830052, China; yan_jiayue@163.com; 2 Key Laboratory of Ministry of Education of Grassland Resources and Ecology in Western Arid Region, College of Grassland Science, Xinjiang Agricultural University, Urümqi 830052, China; 3 Xinjiang Key Laboratory of Grassland Resources and Ecology, College of Grassland Science, Xinjiang Agricultural University, Urümqi 830052, China

**Keywords:** dimorphic diaspore, dispersal, after-ripening, hygroscopic awns, self-burial of seeds, seedling emergence

## Abstract

*Bromus tectorum*, a winter annual plant, produces dimorphic diaspores: complex diaspores with multi-awns and simple diaspores with one awn. However, there is no information available about the role of awns and the germination characteristics of dimorphic diaspores. Dispersal germination and awns hygroscopicity of the dimorphic diaspores were assessed. The complex diaspore with multi-awns can easily be dispersed long distances from the mother plant by mammals. The simple diaspores with one awn are tightly attached to the mother plant. Caryopses from the two types of diaspores exhibited non-deep physiological dormancy at maturity, which can be released by dry storage and GA_3_ treatment. The awns have hygroscopic activity and can move in response to changes in moisture, moving the complex diaspore (the seed) into the soil. The seedling emergence from complex diaspores was significantly higher than those from simple diaspores at all burial depths. Germination of caryopses on the soil surface was poor. The optimal planting depth for both types of diaspores’ emergence is 1–2 cm. The distinct characteristics of dimorphic diaspores and the beneficial influence of hygroscopic awns on dispersal, germination, and seedling establishment have significant ecological implications for *B. tectorum*’s successful reproduction in unpredictable cold deserts.

## 1. Introduction

Climate change has the potential to induce shifts in species ranges and local extinctions, presenting a global threat to plant diversity [1]. For many plant species, dispersed seeds or fruits represent the only mobile stage of the life cycle [2]. The inherent diversity of morphological structures in seeds and fruits arises from diverse strategies employed for successful dispersal and optimal germination timing [3,4]. Seed dispersal helps offspring escape competition with siblings, reduces risks in changing environments across temporal and spatial scales, and allows them to reach safe locations [5,6,7].

Most plant species produce a single type of seed and fruit that is best suited to their respective habitats [8]. Interestingly, many plants have evolved a heterotypic strategy [8,9,10]. In a broad sense, propagator heteromorphism refers to the production of propagators (seeds or single-seeded fruits) with different forms or behaviors by a single individual, including both seed heteromorphism and fruit heteromorphism [11]. Diaspore heteromorphism has been observed in 18 families, most commonly in Compositae, Chenopodiaceae, and Cruciferae [9]. Diverse ecological strategies are associated with diaspore heteromorphism, including differentiation of competitive abilities, dormancy, and dispersal [11,12,13].

From an economic and ecological standpoint, grasses (Poaceae) are the most crucial plant species on Earth [14]. One of the characteristics that is considered a fundamental driver of grassland ecology and evolutionary success is the awn [14,15]. Many grass species allocate significant resources to their awns, which may convey important fitness advantages for the plants [16,17,18]. Proposed dispersal functions include soil burial, epizoochory, and aerial orientation. Awns may also protect the seed from drought, herbivores, or fire by helping it become buried in the soil. In some species, awns function in photosynthesis, providing carbon to the seed [19].

The awn is not only specialized for dispersal but also for mechanical support and directed movement. Hygroscopically active awns of wild wheat (*Triticum turgidum* ssp. *diccocoides*) propel seeds on the ground through coiling and uncoiling [20]. Seed burial depth of *Hyparrhenia diplandra* was influenced by awn length [21]. Peart [22] demonstrates that both depth and orientation of burial significantly impact seed germination. The presence of hygroscopically active awns greatly increases the proportion of seeds positioned favorably within the soil matrix, leading to enhanced germination and seedling survival.

The changes in seed size and dormancy may be attributed to the positioning of the seed within the inflorescence and dispersal unit [23,24]. Diaspores of *Avena sterilis* [25], *Aegilops* species (*A. neglecta*, *A. geniculata* and *A. triuncialis*) [26], and *Eremopyrum distans* [27] contain more than one seed, and the first formed and well-developed seeds have lower dormancy. In *Triticum dicoccoides*, however, the younger upper seed in the spikelet is usually larger and less dormant than the bottom one [28]. Dimorphic seeds located in different positions within the spikelet may serve as a bet-hedging trait allowing a population to survive periods of insufficient rainfall through dormancy [27,28].

*Bromus tectorum* L. is native to the Eurasian continent and the Mediterranean region and is a cleistogamous winter annual plant. Seeds germinate in autumn when there is ample precipitation, and plants mature in late spring [29]. *B. tectorum* produces two types of diaspores on the same spikelet: a complex diaspore (attached with sterile florets) and a simple diaspore (single unit not attached with sterile florets) [30,31]. The caryopsis can be dispersed within a few weeks after maturity through various means such as rain, wind, and different animal vectors [29,31]. The sterile florets promote the dispersal ability of complex diaspores [32]. The seeds of *B. tectorum* show varying degrees of dormancy [33] and have significant differences among populations [34,35,36].

Due to their distinctive roles in plant adaptation to the environment, diaspore polymorphism and awn hygroscopicity are of considerable significance for understanding the evolutionary life-history strategies of grasses. However, information on the role of awns and the germination characteristics of the heteromorphic diaspores of *B. tectorum* is not available.

This study aimed to test the differences in dispersal, dormancy, and germination between dimorphic diaspores, as well as testing the relative humidity dependency of awns and the effects of hygroscopic awns on seed burial and soil depth on seedlings emerging from dimorphic diaspores of *B. tectorum*. The awns could confer a selective advantage. We hypothesized that: (1) complex diaspores with multi-awns will be easier to disperse than simple diaspores with one awn, and dormancy levels may be shallower; (2) hygroscopic awns will propel complex diaspore self-burial; (3) multi-awns will increase diaspores’ water absorption capacity and promote seedling emergence;(4) variation in dimorphic diaspores resulting in dispersal, germination, seed burial, and seedling emergence differences could be adaptive in this annual grass.

## 2. Results

### 2.1. Morphological Characterization of Heteromorphic Diaspores

**Figure 1:**
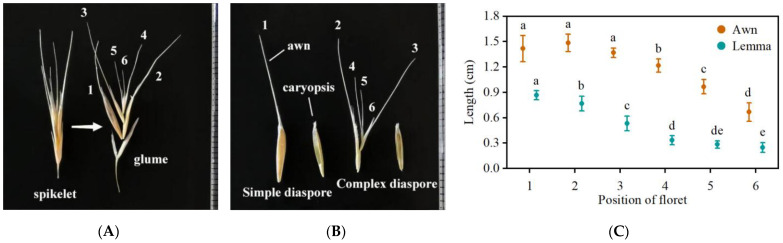
Spikelet composition of *Bromus tectorum*. (**A**) Spikelet consisting of glumes and florets, (**B**) Simple diaspore and complex diaspore. 1 and 2 are fertile caryopses. 3, 4, 5 and 6 are sterile florets. An awn is attached to the top of the lemma. The awn number of a diaspore is equal to the number of sterile florets plus 1. The adjacent bars are 1 mm. (**C**) The length of the awn and lemma of florets at different positions in the spikelet. Different lowercase letters indicate significant difference (Tukey HSD, *p* < 0.05) between the lengths of each awn or lemma across different positions. Error bars = ±1 SE. n = 10.

We investigated the general characteristics of *B. tectorum* reproductive parts and dimorphic diaspores using plants from a natural cold desert population. The inflorescence of *B. tectorum* is a pendant panicle, 15–20 cm in length. The spikelet is 2–4 cm long and consists of 6–8 florets (Figure 1A). The two lower florets are fertile and contain fully developed caryopses, while the upper florets of the spikelet (No. 3–6) are typically sterile and persist attached to the distal caryopsis after maturity. Both fertile and sterile florets possess awns located at the tip of their lemma (Figure 1B). The lengths of the awn and lemma in fertile florets significantly exceeded those of the sterile ones, while in sterile florets, these lengths gradually decreased from the lower to upper positions within the spikelet (Figure 1C).

*B. tectorum* produces complex and simple diaspores. The complex diaspore consists of one caryopsis and several sterile florets, resulting in a diaspore with several awns (Figure 1). The several awns of complex diaspores are oriented in various directions, resulting in a parachute-like shape. The simple diaspores consist of only one caryopsis with one awn and exhibit a slender-tapered shape. The mass of the complex diaspores was significantly greater than that of the simple diaspores (Table 1).

**Figure 2:**
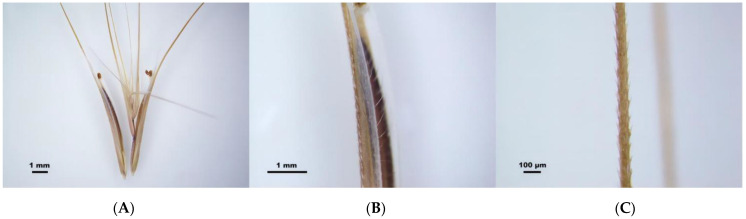
Microhairs and spines on the lemma and awn of *Bromus tectorum* microscopically. (**A**) Diaspores. (**B**) Lemma. Microhairs on the back and margin of the lemma. (**C**) Awn. Spines on the awn.

Under the microscope, there are microhairs on the back and margin of the lemma and silicified hairs and spines on the awns; both the hairs and spines exhibit a consistent orientation along the awns (Figure 2).

### 2.2. Dispersal

We conducted field and controlled experiments and tested dispersal in natural habitats and by mammals.

#### 2.2.1. Dispersal in Natural Habitat

**Figure 3:**
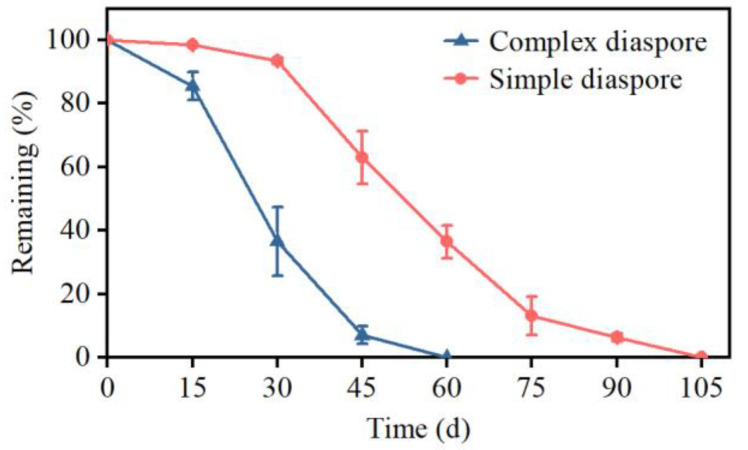
Percentage of diaspores remaining on mother plants of *Bromus tectorum* at 0, 30, 60, 90 and 105 days after diaspore maturity. Error bars = ±1 SE. n = 3 mother plants.

In the 3-month natural dispersal study, 63.4 ± 9.8% and 6.6 ± 1.0% of the complex and simple diaspores, respectively, had dispersed from the plant within 30 days after maturity (Figure 3). Within 60 days after maturity, the complex diaspores were completely dispersed from the mother plant, while 36.5 ± 5.2% of the simple diaspores remained attached.

The highest proportion of dispersal units was found (˃85%) within a 0.3 m radius of the mother plant, and the number of dispersal units significantly decreased as the distance increased.

#### 2.2.2. Dispersal by Mammals

In each meter transect, there were 23.4 diaspores (2344 fruits/100 m) adhering to the mammal model. The attachment of complex diaspores (96.1 ± 2.7%) was significantly higher than that of the complex diaspores (3.9 ± 0.8%) (*p* < 0.05).

### 2.3. Germination

Germination of both types of diaspores was tested at different temperatures. Cold stratification and gibberellic acid (GA_3_) were used to break their dormancy.

#### 2.3.1. Effect of Temperature on Germination

**Figure 4:**
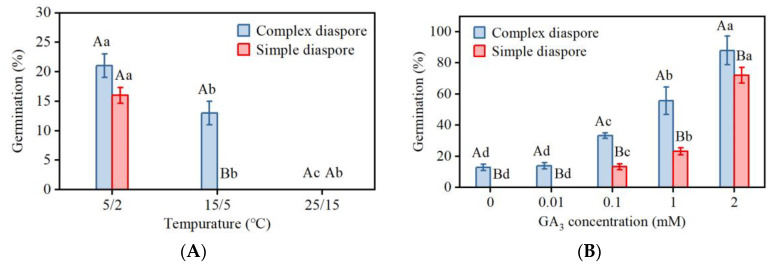
(**A**) Germination percentages of fresh dimorphic diaspores of *Bromus tectorum* at different temperatures. (**B**) Effect of GA_3_ treatment on germination of dimorphic diaspores at 15/5 °C. Different uppercase letters indicate significant difference (*t*-test, *p* < 0.05) between germination percentages of the two different diaspore morphs at the same treatment levels. Different lowercase letters indicate significant difference (Tukey HSD, *p* < 0.05) between germination percentages of the same diaspore morph across different treatments. Error bars = ±1 SE. n = 3 replicates.

Germination of newly matured complex and simple diaspores at various temperatures was <25% and displayed significant variability. With increasing temperature, the germination percentages decreased significantly (Figure 4A). At 25/15 °C, neither of the two types of diaspores germinated, indicating that both were dormant.

#### 2.3.2. Dormant-Break

##### Effect of GA_3_ on Germination

GA_3_ significantly promoted the germination of caryopses. As the concentration of GA_3_ increased, the germination of the complex diaspores and simple diaspores significantly increased (Figure 4B).

##### Effect of Dry Storage (After-Ripening) on Germination 

**Figure 5:**
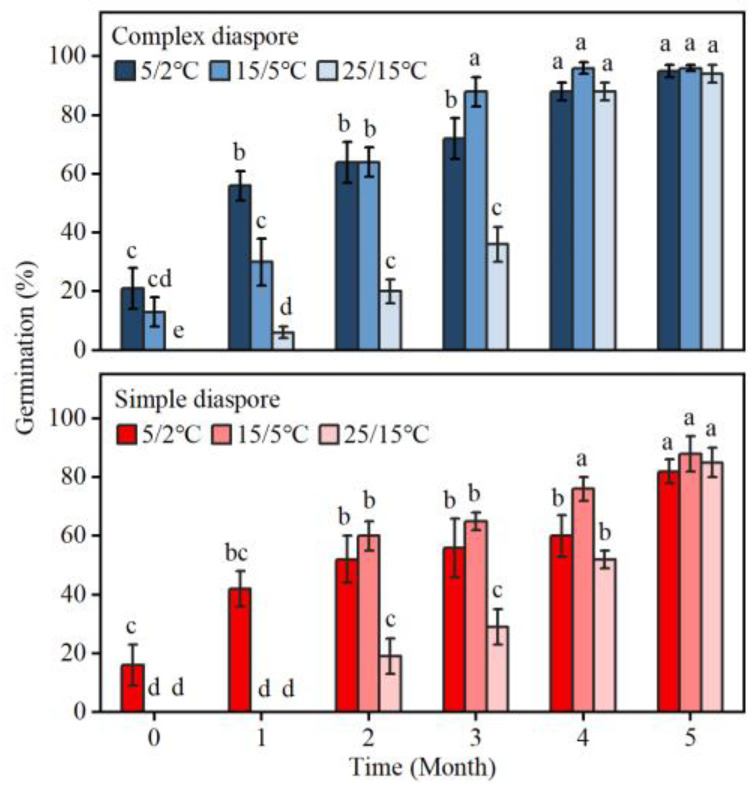
Germination percentages of complex and simple diaspores of *Bromus tectorum* during dry storage. Different lowercase letters indicate significant difference (Tukey HSD, *p* < 0.05) across all times, regimes, and temperature conditions. Error bars = ±1 SE. n = 3 replicates.

With prolonged storage duration, there is a gradual increase in germination. Specifically, germination of complex diaspores was over 80% after 4 months of storage, whereas that of simple diaspores reached this threshold after 5 months (Figure 5).

Furthermore, newly matured diaspores germinated only at low temperatures, and with increasing storage time, the range of germination temperatures gradually expands.

### 2.4. The Role of Awns

#### 2.4.1. Relative Humidity Dependency

**Figure 6:**
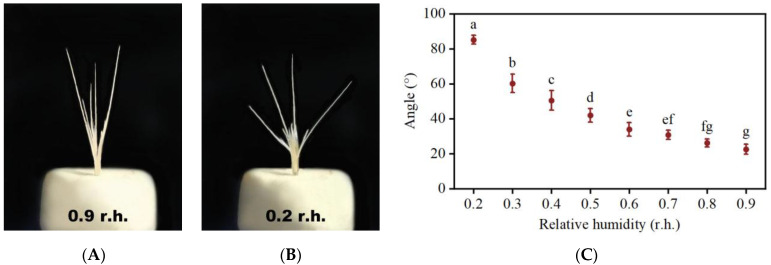
The movement of complex diaspores of *Bromus tectorum*. The relationship between the average angle of complex diaspore awns and the relative humidity (r.h.) of the surrounding air is presented. (**A**) Complex diaspores were exposed to 0.9 r.h. and (**B**) 0.2 r.h. for two hours. (**C**) Effect of relative humidity on angle between the awns. Different lowercase letters indicate significant difference (*p* < 0.05) among different relative humidity.

We conducted controlled relative humidity experiments to test dispersal unit movement. With increasing humidity levels, the awns underwent straightening, resulting in a reduction in the angle between awns (Figure 6A,C). Subsequently, upon drying, the angle between the awns increased (Figure 6B,C). The movement was reversible; therefore, the humidity cycle induced a periodic motion of the awns, potentially resulting in articulation at the base of florets.

#### 2.4.2. Self-Burial of Seeds

After undergoing a 20-day dry-wet cycle, self-burial of complex diaspores was 77.1%, while that of simple diaspores was none. The burial depth of complex diaspores was 0.38 cm.

### 2.5. Water Absorption of Heteromorphic Diaspores

The water absorption process of the two types of diaspores was similar and can be characterized by three distinct stages: a rapid initial water uptake stage, a subsequent stagnation in water uptake, and finally a slow continuous water uptake stage.

**Figure 7:**
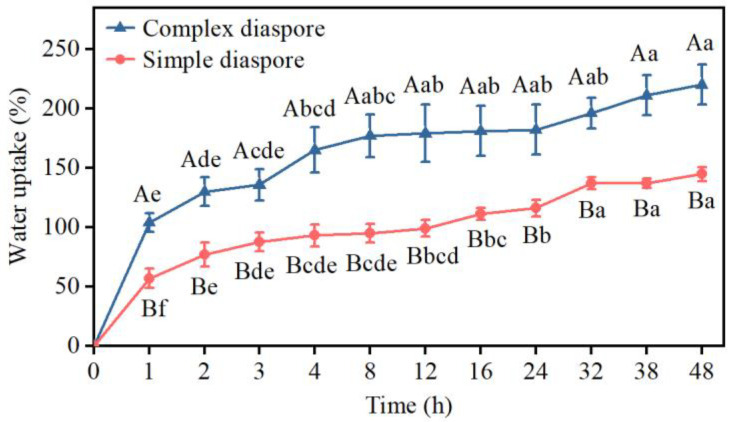
The water uptake curves for dimorphic diaspores of *Bromus tectorum*. Different uppercase letters indicate significant difference (*t*-test, *p* < 0.05) between the two different diaspore morphs at the same times. Different lowercase letters indicate significant difference (Tukey HSD, *p* < 0.05) among different times of the same diaspore morph. Error bars = ±1 SE. n = 3 replicates, each with 100 replicate diaspores.

Throughout the entire process of water absorption, the complex diaspore exhibited a significantly higher water absorption compared to the simple diaspore (*p* < 0.05) (Figure 7). Specifically, within the initial 3 h of contact with water, the complex diaspore and simple diaspore demonstrated rapid water absorption of 135.7% and 87.7%, respectively. Afterwards, water absorption ceased, and subsequently proceeded slowly over a 24 h period, resulting in 220.0% and 144.7% water absorption for complex and simple diaspores, respectively, at 48 h. Following water absorption, the mass of the complex diaspores and simple diaspores increased by factors of 3.2 and 2.4, respectively, compared to their pre-absorption weights.

### 2.6. Effect of Burial Depth on the Germination and Seedling Emergence

**Figure 8:**
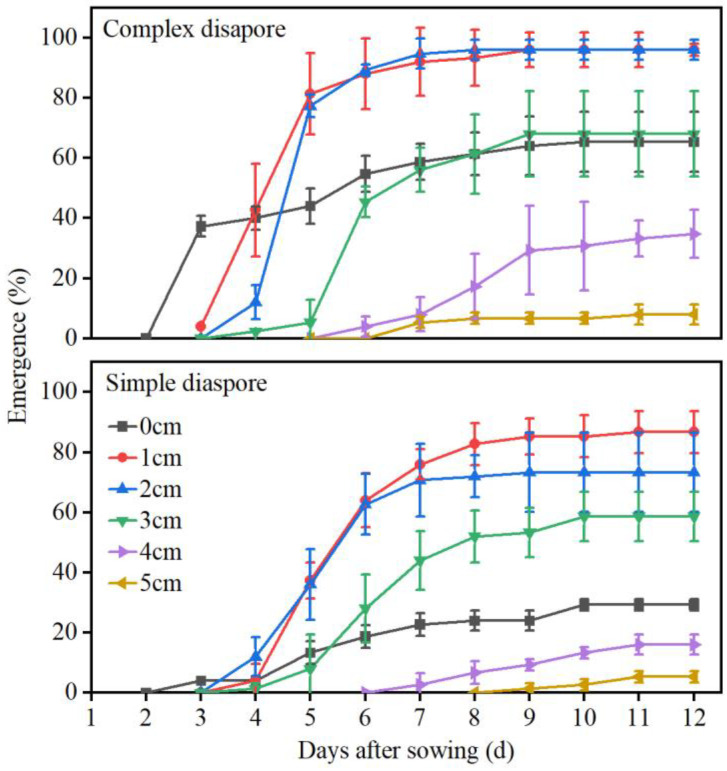
Cumulative emergence of seedlings from complex diaspores and simple diaspores of *Bromus tectorum* planted at varying depths in the soil. Error bars = ±1 SE. n = 3 replicates.

We investigated the impact of burial depth on seedling germination of the two diaspore types through a greenhouse pot experiment. The total germination percentages of two types of caryopses were not significantly affected by planting depths of 1–4 cm, but they decreased significantly at a depth greater than 5 cm. Percentage germination of the two types of diaspores was not significantly affected at all planting depths (Table 2). The diaspores’ germination at 0 cm was significantly lower compared to those with 1–3 cm of soil coverage because the diaspores dried out quickly on the soil surface after being watered. Approximately 65% of the complex diaspores and 29% of the simple diaspores on the surface germinated. The emergence of seedlings exhibited a negative correlation with planting depth (Figure 8). For the two types of diaspores, the highest emergence was observed at planting depths of 1 and 2 cm. A significant reduction in emergence was noted at depths of 3, 4, and 5 cm, while no seedling emergence was observed at depths of 6 and 8 cm. Additionally, the seedling emergence from complex diaspores was significantly higher than that from simple diaspores across all depths. The highest seedling emergence of complex diaspores and simple diaspores was 96.0 ± 2.0% and 86.7 ± 7.0%, respectively, when sown at 1 cm.

## 3. Discussion

### 3.1. Different Dispersal Mechanisms of Dimorphic Diaspores

Seed dispersal is an important part of a plant’s life cycle and is the basis for determining spatial patterns, population growth rates, and species expansion rates [37]. Variations in the morphology of dispersal structures can have profound effects on dispersal in both space and time within a particular species [32,37]. Our biomechanical and morphological comparison of the *B. tectorum* dimorphic diaspores revealed that they correspond to distinct dispersal modes and agents. Whereas monomorphic species are limited to a single dispersal mode for diaspores, heteromorphic species have developed various dispersal and dormancy adaptations as a potential bet-hedging strategy for species survival in unpredictable habitats [4,9,38].

For heteromorphic diaspores, the presence or absence of dispersal structures represents a functionally significant factor [4,9]. In Poaceae, the presence of appendices on one diaspore may be attributed to the abortion of terminal florets, which, if still attached to the most distal fertile caryopsis, may create heteromorphic diaspores [30]. When reaching maturity in early summer, *B. tectorum* diaspores are dispersed by abiotic or biotic agents. Dimorphic diaspores showed different dispersal ability, which is not only dependent on the presence of caryopses with or without sterile florets attached, but also on their position within the spikelet. The upper complex diaspores exhibit a higher tendency to detach from the mother plants compared to the lower simple diaspores. However, the initial dispersal through non-living agents is a highly localized process, as 91% of diaspores land within a 30 cm radius of the parent plants. We consistently observed that complex diaspores had a higher ability to disperse than simple diaspores. Complex diaspores possess multi-awns in multiple directions, each adorned with microhairs and spines, thereby enhancing their adhesive ability to fur. In the presence of mammals, 96% of the diaspores attached to sheep fur are complex diaspores. Awns of *B. tectorum* appear to play a key role in relatively long-distance dispersal [39,40].

### 3.2. Distinct Dormancy and Germination Mechanisms of Dimorphic Diaspores

Seed dormancy is a critical factor in determining the timing of germination and seedling emergence [41,42], impacting the likelihood of seedling survival and the environment for future plant growth [43]. Freshly matured diaspores of *B. tectorum* had low germination, indicating low primary dormancy (Figure 4A). After dry storage (after ripening), the germination percentages were greatly increased (Figure 5). Further, GA_3_ promotes the germination of diaspores (Figure 4B), thus, fresh caryopses of *B. tectorum* have non-deep physiological dormancy [8,44]. In addition, when the germination percentage reached more than 80%, the dry storage time of complex diaspores (4 months) was shorter than that of simple diaspores (5 months), indicating a shallower dormancy level in complex diaspores compared to simple diaspores.

In the challenging and fluctuating environment of the desert, the success of a population hinges on seeds entering dormancy during adverse conditions and germinating during favorable conditions [45]. Dormancy in summer and germination in autumn are characteristic of the seeds of winter annuals [8]. *B. tectorum* caryopses reach maturity in June or even earlier in Junggar Desert habitats. Prolonged periods of high temperatures coupled with intermittent rainfall can elevate the risk of premature summer germination; this hazard, however, can be mitigated by possessing a high primary dormancy and slow reawakening after ripening. Dormancy is terminated during dry storage (after ripening), enabling most seeds to germinate in autumn.

The optimal germination is highly responsive to variations in dispersibility, particularly within the constrained range of dispersibilities that are typical for annual plants. As dispersibility rises, the optimal germination fraction also increases [46,47]. If plants produce two types of seeds with varying dispersal abilities, the optimal reproductive strategy involves seeds with low dispersal delaying germination (having dormancy) and seeds with high dispersal germinating quickly (having non-dormancy) [38,46]. In some grasses, seeds differing in their position within a dispersal unit or spikelet often vary in size and dormancy [28,48,49]. Our experiments suggest that the dormancy level of the upper complex diaspores, which are easily dispersed by animals over long distances, is lower than that of the lower simple diaspores (Figure 5). The simple diaspores may form a better soil seed bank than the complex diaspores. In *B. tectorum*, two distinct types of diaspores maybe potentially increase the survival chance of its offspring in time- and space-variable environments.

### 3.3. The Hygroscopic Movement of Awns, Self-Burial of Seeds, and Seedling Emergence

Many grass species produce hygroscopic awns that mechanically circulate with changes in air moisture [50]. Mechanical pressure caused by the hygroscopic movement of the awns pushes the seeds into the ground [22,51]. Possessing well-developed awns confers an ecological advantage by increasing the likelihood of seeds finding micro-sites favorable for germination and establishment [22,50].

The awns of *B. tectorum* have humidity-dependent motion. After undergoing a 20-day dry-wet cycle, the motion of awns makes complex diaspores self-bury to a depth of 0.3 cm. In the Junggar desert, there is a humidity cycle through the dispersal period, during which the air is dry during the day, but humidity increases at night as temperatures decrease. We have also observed that the unidirectional movement of diaspores results in deeper burial in the soil. The humidity-driven movement of the awns has also been observed in *Avena sterilis* [52], *Triticum turgidum* ssp. *Diccocoides* [19], and *Stipa epilosa* [53]. In fire-prone habitats, seeds with hygroscopic awns are often buried a few centimeters below the soil surface, which minimizes the risk of exposure to high temperatures that might kill the seeds [21,22,54]. In *Triticum turgidum* ssp. *diccocoides*, the bending of the awns is attributed to the arrangement of cellulose fibrils and its response to changes in humidity [19]. The mechanism behind the bending of awns in sterile florets of *B. tectorum* with respect to humidity warrants further investigation.

Seedling emergence and establishment are influenced by seed burial depth [8,55]. In both types of *B. tectorum* diaspores, seed burial at 1–2 cm led to the greatest seedling emergence percentages. The seedling emergence for seeds at 0 cm burial depth (located on the soil surface) decreased compared with that at 1 cm burial depth, which might be attributed to the reduced water availability because of limited soil contact [56]. It appears that a minimal soil coverage of a few millimeters is necessary for optimal germination and emergence. The seedling emergence for complex diaspores at 0 cm burial depth was higher than that of the simple diaspores (Table 2), which may be a result of the larger water absorption of the complex diaspores compared to the simple diaspores (Figure 7). The seedling emergence of the complex diaspores was greater than that of the simple diaspores at 3–5 cm burial depth (Figure 8). This outcome may be a result of the larger seed size of the complex diaspores compared to that of simple diaspores. Non-emergent seeds had germinated but failed to emerge. This may be attributed to the insufficient nutritional supply for the shoots to reach the soil surface when seeds are buried at greater depths [8,57,58].

*B. tectorum* is one of the most destructive invasive annual plants in North American dryland ecosystems [59]. *B. tectorum*’s diaspore dimorphism and awn hygroscopicity may synergize with future climate conditions and increase its competitive advantage over native plants, as *B. tectorum* may be able to take advantage in seed dispersal, seed burial, and seedling establishment.

## 4. Materials and Methods

### 4.1. Source of Spikelets (With Fully Developed Caryopsis)

**Figure 9:**
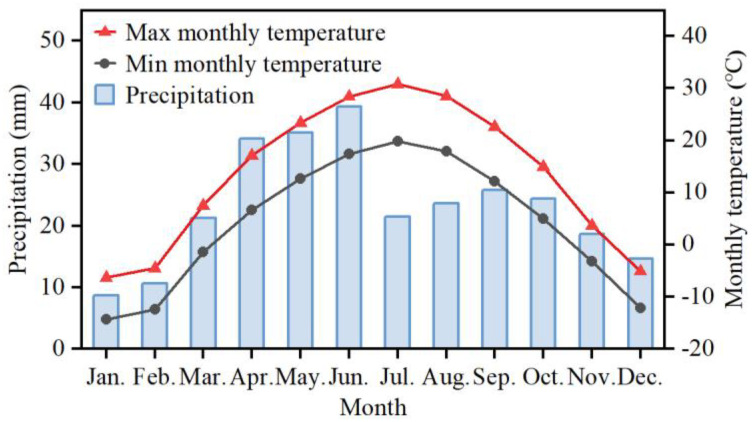
Mean maximum, mean minimum, and mean monthly temperature in Urumqi, Xinjiang, China (2013–2023). Temperature and rainfall data were obtained from the local government.

Freshly matured spikelets of *B. tectorum* were collected on 29 May 2023 from 50 individual plants in a natural population in the cold desert in the Junggar Basin in Xinjiang (NW China) (43°43′38″ N, 87°27′19″ E, 1120 m a.s.l.). The plants were spaced 0.5 m apart. *B. tectorum* was the dominant species at the site, and it grew with *Medicago lupulina*, *Neotorularia korolkovii*, *Lepidium ruderale*, and other species. This area exhibits a typical continental semi-arid/arid climate with annual precipitation ranging from 220 to 270 mm. Winter precipitation accumulates as snowfall, which subsequently melts during spring. Based on the data from Xinjiang Meteorological Information Center covering the period from 2013 to 2023, the mean temperature during the warmest month (July) is 30.8 °C, and during the coldest month (January) is −14.4 °C (Figure 9).

In Junggar Basin, *B. tectorum* germinates in autumn (October), overwinters under snow, and reaches maturity in late spring (late May or early June). The harvested spikelets (caryopses) were air-dried at room temperature (20–25 °C, 20–35% relative humidity) for 1 week before initiation of germination tests for fresh seeds, dormancy breaking, and sown in pots. The two types of diaspores were used in this study.

### 4.2. Morphology Chacteristics of Diaspores

In the laboratory, a stereomicroscope was used to observe the composition of the spikelets and the position of fully developed caryopsis and sterile florets. The florets in the spikelets were numbered from the bottom to the tip (1 to 7), with 1 being the lowest (proximal) flower on the spikelet and 7 being the highest (distal) flower. In this study, the diaspore was used to describe the dispersal unit of *B. tectorum* composed of a single caryopsis, including the lemma, palea, and caryopsis. The mass of both types of diaspores and caryopses was measured using a Sartorius balance (0.0001 g), with 4 replicates conducted for 100 grains.

We utilized a Nikon digital stereomicroscope (SMZ1000) for the observation and photography of diaspores, caryopses, and the lemma and awn, and employed Image J (v.1.54) for quantifying their dimensions. We also conducted an analysis of the orientation and distribution of appendant structures (hairs, spines) on the lemma and awn.

### 4.3. Dispersal Experiment

#### 4.3.1. Abiotic Dispersal: Detachment of Diaspores from Mother Plants in Natural Habitat

To determine the time and distance of the diaspores’ fall from the parent plant, three plants were randomly selected (at least 30 m apart) and each was enclosed within a square frame (1 m × 1 m × 1 m) covered with a transparent plastic mesh (mesh size 2 mm × 2 mm) to isolate the plants before the first diaspore was dispersed (early June 2022). A piece of cotton fabric was placed on the soil surface and fixed with anchor pins. In this manner, the diaspores that landed on it became affixed to the cotton fabric. Monitoring began from the spikelets’ ripening (15 June 2023) to the end of dispersal (September 2023). Every 15 days, a meter tape was used to determine the distance. Subsequently, all dispersal units that had landed on the cotton fabric were collected and the number of both types of diaspores was quantified.

#### 4.3.2. Biotic Dispersal: Epizoochory

To assess the potential for mammals to act as carriers, we employed a method based on Baker and O’Dowd [60] and Mouissie et al. [39] by encasing a PVC pipe (27 cm in length, 10 cm in diameter) with sheep wool to simulate a small mammal model representing part of a sheep’s body. The mammal model was secured by a rope and systematically traversed 10 transects of 10 m through plant populations in June. Subsequently, the number of both types of diaspores sticking to the mammal model was counted.

### 4.4. Germination Experiments

Experiments on germination were carried out using three replicates with 25 diaspores of each of the two diaspore morphs. The diaspores were placed on a double layer of moist filter paper in a 9 cm Petri dish, covered with an additional layer of filter paper, and then 15 mL of distilled water was added to ensure complete absorption. Subsequently, all the Petri dishes were sealed with cling film and put into an illuminated incubator for 14 days.

#### 4.4.1. Effect of Temperature on the Germination of Heteromorphic Diaspores

Freshly matured simple diaspores and complex diaspores (1 weeks after collection) were placed in incubators at 5/2 °C, 15/5 °C, and 25/15 °C (light 12 h/dark 12 h). The fluctuating temperature ranges approximately represent the mean daily maximum and minimum air temperatures during the seasons in the Urumqi west mountain desert of Junggar Basin: March and November, 5/2 °C; April, May and October, 15/5 °C; July, August and September, 25/15 °C.

#### 4.4.2. Dormancy-Break

The germination percentages of freshly matured simple diaspores and complex diaspores were less than 30% (see result), showing that the caryopses of two types of diaspores were dormant. Thus, the effects of GA_3_ and dry storage were tested on dormant release. 

##### GA_3_ Treatments

To assess the sensitivity of dimorphic diaspores to GA_3_, diaspores of each type were planted on Petri dishes containing 0, 0.01, 0.1, 1.0, and 2.0 mM of GA_3_ for germination experiments at 15/5 °C (GA_3_ was first dissolved in ethanol and then diluted with distilled water to the desired volume).

##### Dry Storage (After-Ripening)

Simple diaspores and complex diaspores stored in laboratory conditions for 0 (fresh), 1, 2, 3, 4 and 5 months were then tested for germination at 5/2 °C, 15/5 °C, and 25/15 °C, as described above.

### 4.5. Awns Hygroscopic Movement and Diaspore Burial

#### 4.5.1. Awn Hygroscopicity

The air’s relative humidity was controlled within a range of 0.2–0.9 using a humidity chamber at 25 °C. The diaspores (only tested with complex diaspores) were vertically positioned in the rubber mud at a depth of 3 mm to achieve stability, and the movement of the diaspores was recorded within the constant humidity chamber. Following exposure to consistent humidity conditions for at least two hours, digital photographs were taken and the angle between the awns of complex diaspores was measured using an Image J (v.1.54).

#### 4.5.2. Burial Depth in the Soil

Referring to the method of Garnier and Dajoz [21], eight rectangular pots (20 × 30 × 10 cm) were filled with native soil, manually crushed, and compacted. For both types of diaspores, four replicates (pots) of 25 diaspores were arranged in a vertical position with their bases partially buried in the soil (3 mm) for stability and spaced at 3 cm intervals in all directions from each other. For a period of 20 days, each replicate was sprayed with water mist every morning to stimulate the movement of the hygroscopic awns. Subsequently, the awns were treated with red paint spray to mark the exposed part of the diaspore. This ensured that the buried part of the diaspore was uncolored and could be measured using a digital caliper under magnification.

### 4.6. Water Uptake

To investigate the process of water absorption and multi-awns influence on diaspores’ water absorption capacity, diaspores with a 5-month dry storage period were chosen for the experiment to ensure complete release of seed dormancy. Simple diaspores and complex diaspores were placed, respectively, in Petri dishes lined with two layers of filter paper, moistened with 15 mL of distilled water, and covered with an additional layer of filter paper to facilitate complete water absorption. At specific time intervals (0, 1, 2, 3, 4, 8, 12, 16, 24, 32, 38, and 48 h), diaspores were collected from the dishes and subsequently desiccated with filter paper to remove excess moisture and weighed with a Sartorius balance (0.0001 g). The diaspores were then returned to the Petri dishes until the first seed germinated. Three replicates were undertaken with 100 diaspores. The percentage of water absorbed was determined by dividing the increase in weight by the initial weight.

### 4.7. Effect of Burial Depth on Germination and Seedling Emergence

Two types of diaspores were sown in a greenhouse in October 2023 (the time of year when germination occurs under natural conditions). Seven distinct burial depths (0, 1, 2, 3, 4, 5, 6 and 8 cm) were duplicated in three sets with 25 diaspores per set. The pots (diameter 15 cm) were marked approximately 2 cm from the upper rim. Another marking was placed on the same side at the specified burial depth for the caryopses. Each pot was equipped with a single drainage outlet shielded by nylon mesh to mitigate soil loss and enhance excess water drainage, while the soil was filled up to the lower marking. The caryopses were positioned on the surface of the soil, and then soil was filled to reach the upper marking and moistened until it attained field capacity. The pots were watered gently with a fine mist every day.

Seedling emergence was recorded daily until approximately 12 days after planting, when seedlings ceased emerging. The contents of each pot were carefully poured out to ensure the caryopses’ as well as the emerged or unemerged seedlings’ intactness. The diaspores and seedlings from each treatment underwent careful examination using forceps and microscopic observation to assess their condition.

### 4.8. Statistical Analysis

The mean ± standard error was used to express all data. ANOVA followed by a *t*-test was employed for paired independent samples to assess differences in morphology and mass, number, and percentage of the two types of diaspores remaining on the mother plants, the percentage of attached dispersal units on the surface of the mammal model, and the mass of water absorbed between the two diaspore morphs. Tukey’s HSD test was performed for multiple comparisons to analyze data collected from germination experiments regarding temperature, dry storage, GA_3_ application, and burial depths. Statistical tests were conducted at a significance level of *p* = 0.05 using SPSS 26.0 software (SPSS Inc., Chicago, IL, USA).

## 5. Conclusions

The dimorphic dispersal units of *B. tectorum* exhibit differences in morphology and diaspore and germination ecology. The non-deep physiological dormancy (after ripening) prevents seeds from germinating in the summer, but seeds are nondormant by autumn and can germinate at that time if the soil is moist. The multi-awns of complex diaspores increase dispersal distances by sheep. The awns of sterile florets exhibit hygroscopic properties, facilitating the movement of complex diaspores into the ground and promoting seed emergence. Diaspore dimorphism and awn hygroscopicity can help locate seeds in safe sites for germination and improve seedling establishment, which permits this species’ successful survival in the harsh cold desert. This is especially important for increasingly unpredictable environments, which climate change is making more common.

## Figures and Tables

**Table 1:** The morphology characteristic of the two diaspore types of *Bromus tectorum.*

Type	Shape	No. of Awn	Length (cm)	Diaspore Mass (mg)	Seed Mass (mg)
Complex diaspore	Parachute	4–6 a	2.47 ± 0.15 a	1.79 ± 0.13 a	1.27 ± 0.02 a
Simple diaspore	Slim-taper	1 b	2.13 ± 0.04 a	1.27 ± 0.07 b	1.17 ± 0.03 a

Values with different lowercase letters in a column denote significant different the two different diaspore morphs (*t*-test, *p* < 0.05).

**Table 2:** Germination and seedling emergence of two types of artificially buried diaspores of *Bromus tectorum* planted in pots at varying depths in the soil.

Depth (cm)	Complex Diaspore			Simple Diaspore		
	Germination (%)	Emerged Seedling (%)	Unemerged Seedling (%)	Germination(%)	Emerged Seedling (%)	Unemerged Seedling (%)
0	65.3 ± 10.0 Ac	65.3 ± 10.0 Ab	0 Ad	29.3 ± 1.9 Bc	29.3 ± 1.0 Bc	0 Ad
1	100 Aa	96.0 ± 2.0 Aa	4 ± 0.2 Ad	99.0 ± 1.0 Aa	86.7 ± 7.0 Ba	12.3 ± 3.2 Ac
2	100 Aa	96.0 ± 3.3 Aa	4 ± 0.2 Ad	83.7 ± 10.1 Aa	73.3 ± 13.2 Ba	10.4 ± 2.4 Ac
3	88.0 ± 6.8 Aa	68.0 ± 14.2 Ab	20 ± 4.3 Ac	84.0 ± 9.1 Aba	58.7 ± 8.2 Bb	25.3 ± 3.5 Ab
4	88.0 ± 8.2 Aa	34.7 ± 8.0 Ac	53.3 ± 7.5 Bb	88.0 ± 7.9 Aa	16 ± 3.3 Bc	72.0 ± 10.3 Aa
5	78.0 ± 7.4 Ab	8.0 ± 3.3 Ad	70.0 ± 9.1 Aa	76 ± 8.3 Ab	5.3 ± 1.9 Ad	71.7 ± 6.7 Aa
6	75.1 ± 4.2 Ab	0 Ad	75.1 ± 4.2 Aa	72.0 ± 5.1 Ad	0 Ad	72.0 ± 5.1 Aa
8	73.4 ± 3.7 Ab	0 Ad	73.4 ± 3.7 Aa	71.8 ± 4.2 Ad	0 Ad	71.8 ± 4.2 Aa

Values in a line followed by the different uppercase letter are significantly different between the same characteristic (germination/emerged seedling/unemerged seedling) of the two different diaspore morphs at the same depth (*t*-test, *p* < 0.05). Values in a column followed by the different lower letter are significantly different among different depths (Tukey HSD, *p* < 0.05).

## Data Availability

The original contributions presented in the study are included in the article, further inquiries can be directed to the corresponding authors.

## References

[1] Walck J.L., Hidayati S.N., Dixon K.W., Thompson K., Poschlod P. (2011). Climate change and plant regeneration from seed. Global Change Biol..

[2] Kesseler R., Stuppy W. (2012). Seeds: Time Capsules of Life.

[3] Steinbrecher T., Leubner-Metzger G. (2017). The biomechanics of seed germination. J. Exp. Bot..

[4] Arshad W., Sperber K., Steinbrecher T., Nichols B., Jansen V.A.A., Leubner-Metzger G., Mummenhoff K. (2019). Dispersal biophysics and adaptive significance of dimorphic diaspores in the annual *Aethionema arabicum* (Brassicaceae). New Phytol..

[5] Venable D.L., Brown J.S. (1993). The population-dynamic functions of seed dispersal. Vegetatio.

[6] Levin S.A., Muller-Landau H.C., Nathan R., Chave J. (2003). The ecology and evolution of seed dispersal: A theoretical perspective. Annu. Rev. Ecol. Evol. Syst..

[7] Venable D.L., Flores-Martinez A., Muller-Landau H.C., Barron-Gafford G., Becerra J.X. (2008). Seed dispersal of desert annuals. Ecology.

[8] Baskin C.C., Baskin J.M. (2014). Seeds: Ecology, Biogeography, and Evolution of Dormancy and Germination.

[9] Imbert E. (2002). Ecological consequences and ontogeny of seed heteromorphism. Perspect. Plant Ecol. Evol. Syst..

[10] Song J., Wang H., Chu R., Zhao L., Li X., An S., Qiang M., Du W., Li Q. (2023). Differences in physiological characteristics, seed germination, and seedling establishment in response to salt stress between dimorphic seeds in the Halophyte *Suaeda liaotungensis*. Plants.

[11] Venable D.L. (1985). The evolutionary ecology of seed heteromorphism. Am. Nat..

[12] Mandak B. (1997). Seed heteromorphism and the life cycle of plants: A literature review. Preslia.

[13] Scholl J.P., Calle L., Miller N., Venable D.L. (2020). Offspring polymorphism and bet hedging: A large-scale, phylogenetic analysis. Ecol. Lett..

[14] Linder H.P., Lehmann C.E.R., Archibald S., Osborne C.P., Richardson D.M. (2018). Global grass (Poaceae) success under pinned by traits facilitating colonization, persistence and habitat transformation: Grass success. Biol. Rev..

[15] Humphreys A.M., Antonelli A., Pirie M.D., Linder H.P. (2011). Ecology and evolution of the diaspore “burial syndrome”. Evolution.

[16] Adams K.M., Tainton N.M. (1990). The function of the hygroscopic awn of *Themeda triandra*. J. Grassl. Soc. S. Afr..

[17] Cavanagh A.M., Godfree R.C., Morgan J.W. (2019). An awn typology for Australian native grasses (Poaceae). Aust. J. Bot..

[18] Ansong M., Pickering C. (2014). Weed seeds on clothing: A global review. J. Environ. Manag..

[19] Petersen K.B., Kellogg E.A. (2022). Diverse ecological functions and the convergent evolution of grass awns. Am. J. Bot..

[20] Elbaum R., Zaltzman L., Burgert I., Fratzl P. (2007). The role of wheat awns in the seed dispersal unit. Science.

[21] Garnier L.K.M., Dajoz I. (2001). Evolutionary significance of awn length variation in a clonal grass of fire-prone savannas. Ecology.

[22] Peart M.H. (1984). The effects of morphology, orientation and position of grass diaspores on seedling survival. J. Ecol..

[23] Gosling P.G., Butler R.A., Black M., Chapman J.M. (1981). The onset of germination ability in developing wheat. J. Exp. Bot..

[24] Gutterman Y. (1993). Seed Germination in Desert Plants.

[25] Volis S. (2014). Dormancy-related seed positional effect in two populations of an annual grass from locations of contrasting aridity. PLoS ONE.

[26] Maranon T. (1989). Variations in seed size and germination in three *Aegilops* species. Seed Sci. Technol..

[27] Wang A.B., Tan D.Y., Baskin C.C., Baskin J.M. (2010). Effect of seed position in spikelet on life history of *Eremopyrum distans* (Poaceae) from the cold desert of north-west China. Ann. Bot..

[28] Volis S. (2016). Seed heteromorphism in *Triticum dicoccoides*: Association between seed positions within a dispersal unit and dormancy. Oecologia.

[29] Klemmedson J.O., Smith J.G. (1964). Cheatgrass (*Bromus tectorum*). Bot. Rev..

[30] McKone M.J. (1985). Reproductive biology of several bromegrasses (*Bromus*) breeding system, pattern of fruit maturation, and seed set. Am. J. Bot..

[31] Monty A., Brown C.S.C., Johnston D.B.D. (2013). Fire promotes downy brome (*Bromus tectorum* L.) seed dispersal. Biol. Invasions.

[32] Monty A., Maebe L., Mahy G., Brown C.S. (2016). Diaspore heteromorphism in the invasive *Bromus tectorum* L. (Poaceae): Sterile florets increase dispersal propensity and distance. Flora.

[33] Gasch C., Bingham R. (2006). A study of *Bromus tectorum* L. seed germination in the Gunnison Basin, Colorado. BIOS.

[34] Beckstead J., Meyer S.E., Allen P.S. (1996). *Bromus tectorum* seed germination: Between-population and between-year variation. Can. J. Bot..

[35] Meyer S.E., Allen P.S., Beckstead J. (1997). Seed germination regulation in *Bromus tectorum* (Poaceae) and its ecological significance. Oikos.

[36] Meyer S.E., Allen P.S. (1999). Ecological genetics of seed germination regulation in *Bromus tectorum* L. I. Phenotypic variance among and within populations. Oecologia.

[37] Cousens R., Dytham C., Law R. (2008). Dispersal in Plants, a Population Perspective.

[38] Baskin J.M., Lu J.J., Baskin C.C., Tan D.Y., Wang L. (2014). Diaspore dispersal ability and degree of dormancy in heteromorphic species of cold deserts of northwest China: A review. Perspect. Plant Ecol. Evol. Syst..

[39] Mouissie A.M., Lengkeek W., Diggelen R.V. (2005). Estimating adhesive seed dispersal distances: Field experiments and correlated random walks. Funct. Ecol..

[40] Tackenberg O., Römermann C., Thompson K., Poschlod P. (2006). What does diaspore morphology tell us about external animal dispersal? Evidence from standardized experiments measuring seed retention on animal-coats. Basic Appl. Ecol..

[41] Batlla D., Ghersa C.M., Benech-Arnold R.L. (2020). Dormancy, a critical trait for weed success in crop production systems. Pest Manag. Sci..

[42] Jia C.Z., Wang J.J., Chen D.L., Hu X.W. (2022). Seed germination and seed bank dynamics of *Eruca sativa* (Brassicaceae): A weed on the northeastern Edge of Tibetan Plateau. Front. Plant Sci..

[43] Donohue K., de Casas R.R., Burghardt L., Kovach K., Willis C.G. (2010). Germination, postgermination adaptation, and species ecological ranges. Annu. Rev. Ecol. Evol. Syst..

[44] Baskin C.C., Baskin J.M. (2020). Breaking seed dormancy during dry storage: A useful tool or major problem for successful restoration via direct seeding?. Plants.

[45] Freas K.E., Kemp P.R. (1983). Some relationships between environmental reliability and seed dormancy in desert annual plants. J. Ecol..

[46] Venable D.L., Lawlor L. (1980). Delayed germination and dispersal in desert annuals: Escape in space and time. Oecologia.

[47] Venable D.L., Brown J.S. (1988). The selective interactions of dispersal, dormancy, and seed size as adaptations for reducing risk in variable environments. Am. Nat..

[48] Gianella M., Balestrazzi A., Pagano A., Müller J.V., Kyratzis A.C., Kikodze D., Canella M., Mondoni A., Rossi G., Guzzon F. (2019). Heteromorphic seeds of wheat wild relatives show germination niche differentiation. Plant Biol..

[49] Wang A.B., Baskin C.C., Baskin J.M., Ding J. (2022). Seed position in spikelet as a contributing factor to the success of the winter annual invasive grass *Aegilops tauschii*. Front. Plant Sci..

[50] Cavanagh A.M., Morgan J.W., Godfree R.C. (2020). Awn morphology influences dispersal, mMicrosite selection and burial of Australian native grass diaspores. Front. Ecol. Evol..

[51] Ghermandi L. (1995). The effects of the awn on the burial and germination of *Stipa speciosa* (Poaceae). Acta Oecol..

[52] Somody C.N., Nalewaja J.D., Miller S.D. (1985). Self-burial of wild oat florets. Agron. J..

[53] Yanez A., Desta I., Commins P., Magzoub M., Naumov P. (2018). Morphokinematics of the hygroactuation of feather grass awns. Adv. Biosyst..

[54] Cheplick G.P., Sung L.Y. (1998). Effects of maternal nutrient environment and maturation position on seed heteromorphism, germination, and seedling growth in *Triplasis purpurea* (Poaceae). Int. J. Plant Sci..

[55] Amini R., Ebrahimi A., Dabbagh M.N.A. (2021). Germination and emergence of *Astrodaucus orientalis* (L.) Drude populations influenced by environmental factors and seed burial depth. Plant Species Biol..

[56] Amini R., Gholami F., Ghanepour S. (2017). Effects of environmental factors and burial depth on seed germination and emergence of two populations of *Caucalis platycarpos*. Weed Res..

[57] Wu X., Li J., Xu H., Dong L. (2015). Factors affecting seed germination and seedling emergence of Asia Minor Bluegrass (*Polypogon fugax*). Weed Sci..

[58] Zhao N., Li Q., Guo W., Zhang L., Ge L., Wang J. (2018). Effect of environmental factors on germination and emergence of Shortawn Foxtail (*Alopecurus aequalis*). Weed Sci..

[59] Knapp P.A. (1996). Cheatgrass (*Bromus tectorum* L) dominance in the Great Basin Desert: History, persistence, and influences to human activities. Glob. Environ. Change.

[60] Baker G.A., O’Dowd D.J. (1982). Effects of parent plant density on the production of achene types in the annual *Hypochoeris glabra*. J. Ecol..

